# Taxonomic and functional β-diversity patterns reveal stochastic assembly rules in microbial communities of seagrass beds

**DOI:** 10.3389/fpls.2024.1367773

**Published:** 2024-02-28

**Authors:** Xiaofeng Niu, Wenjing Ren, Congjun Xu, Ruilong Wang, Jingwei Zhang, Huan Wang

**Affiliations:** ^1^ School of Marine Biology and Fisheries, State Key Laboratory of Marine Resource Utilization in South China Sea, Hainan University, Haikou, Hainan, China; ^2^ Institute of Hydrobiology, Chinese Academy of Sciences, Wuhan, Hubei, China

**Keywords:** stochastic assembly, β-diversity, replacement, attached microbial communities, seagrass bed, habitat fragmentation

## Abstract

Microorganisms are important members of seagrass bed ecosystems and play a crucial role in maintaining the health of seagrasses and the ecological functions of the ecosystem. In this study, we systematically quantified the assembly processes of microbial communities in fragmented seagrass beds and examined their correlation with environmental factors. Concurrently, we explored the relative contributions of species replacement and richness differences to the taxonomic and functional β-diversity of microbial communities, investigated the potential interrelation between these components, and assessed the explanatory power of environmental factors. The results suggest that stochastic processes dominate community assembly. Taxonomic β-diversity differences are governed by species replacement, while for functional β-diversity, the contribution of richness differences slightly outweighs that of replacement processes. A weak but significant correlation (*p* < 0.05) exists between the two components of β-diversity in taxonomy and functionality, with almost no observed significant correlation with environmental factors. This implies significant differences in taxonomy, but functional convergence and redundancy within microbial communities. Environmental factors are insufficient to explain the β-diversity differences. In conclusion, the assembly of microbial communities in fragmented seagrass beds is governed by stochastic processes. The patterns of taxonomic and functional β-diversity provide new insights and evidence for a better understanding of these stochastic assembly rules. This has important implications for the conservation and management of fragmented seagrass beds.

## 1 Introduction

Assembly mechanisms are closely linked to changes in community structure, diversity, and the ecological functions they support (Fukami, 2015). This phenomenon has been extensively studied in various ecosystems, such as soil microbes (Wang et al., 2022; Cao et al., 2023), bacterioplankton (Dai et al., 2017), intertidal communities (Loke and Chisholm, 2023), and plants (Peter and Harrington, 2018; Tanunchai et al., 2023). Assembly processes are influenced by various environmental factors, with community turnover cycles limiting exploration. Microorganisms, which are often the most abundant group within systems, play a crucial role in material and energy cycling (Tarquinio et al., 2019; Lemay et al., 2021; Nguyen et al., 2022). Due to their short life cycles, rapid community turnover, and sensitivity to the environment, they are excellent subjects for investigating community assemblage (Vogel et al., 2021a, Vogel et al., 2021b). Seagrass bed ecosystems are known for their highly diverse habitats and host abundant microbial communities, providing habitats for various marine organisms and numerous ecosystem services (Yarnall et al., 2022; Han et al., 2023). These characteristics make seagrass beds an ideal environment for studying microbial community assembly mechanisms (Santos et al., 2016; Du et al., 2020). However, due to factors like climate change, pollution influx, and commercial fishing, seagrass beds in China are rapidly declining (Santos et al., 2016; Xu et al., 2022), resulting in significant losses of ecosystem services they provide (Xiao et al., 2020; Li et al., 2022). Despite ongoing attention and research on related issues, our comprehension of microbial community assembly in fragmented seagrass beds remains limited (Du et al., 2020; Hu et al., 2021), constraining our ability to develop applications for seagrass bed conservation and restoration.

The widely embraced null model method, which quantifies community assembly using beta-mean nearest taxon distance (β-NTI) based on phylogenetic distance and species abundance (Stegen et al., 2012), is designed to elucidate spatial alterations in phylogenetic β-diversity (Zhou and Ning, 2017). Assembly processes, which encompass deterministic and stochastic components, provide insights into variations in community structure composition and diversity dynamics (Prosser et al., 2007). Currently, the latter has garnered significant attention and research focus in seagrass habitats (Crump et al., 2018; Liu et al., 2022; Kardish and Stachowicz, 2023). However, investigations into community assembly, particularly concerning microbial communities, remain relatively limited (Leibold et al., 2017; Zhou and Ning, 2017; Fahrig, 2019). Understanding changes in microbial communities is crucial for seagrass growth (Milbrandt et al., 2008), health (Vandenkoornhuyse et al., 2015), and the ecological functions they provide (Liu et al., 2017; Zhang et al., 2023). Additionally, related research indicates that microbial communities are seldom uniformly distributed in the environment and their spatial variation is primarily dependent on diffusion (Vincent et al., 2021). However, it is currently unknown how microbial function varies as a result.

β-diversity refers to the variations in species composition between different communities, delineated by the processes of species replacement and richness differences. Methods for partitioning β-diversity aim to discern the roles of these processes in total β-diversity, investigating their collective impact on species distribution patterns across diverse spatiotemporal dimensions (Baselga, 2010; Podani and Schmera, 2011). Both taxonomic and functional β-diversity can be quantified between communities, offering valuable insights into the rules governing micro-community assembly (Luo et al., 2023). However, analysis of taxonomic data is inherently limited as it does not provide information about the ecological roles of species (McGill et al., 2006). The use of functional data aids in obtaining a more nuanced comprehension of biodiversity patterns and processes (Swenson et al., 2012; Villéger et al., 2012), which is a well-established application in related research (Swenson et al., 2011; Cardoso et al., 2014).

Since the introduction of β-diversity patterns (Williams, 1996; Lennon et al., 2001), the diversity patterns of plant and animal species have been extensively studied, resulting in various hypotheses (Shen et al., 2020). More recently, attention has also been given to microbial communities, particularly in soils (Li et al., 2020), with fewer investigations in aquatic environments (Sun et al., 2024). However, there is still a lack of understanding regarding fragmented seagrass bed habitats. These patches may resemble individual islands, but their dynamics are significantly influenced by the ebb and flow of seawater. Therefore, classical island ecology theories, such as the area-diversity theory, may not be directly applicable to seagrass bed patches (Chisholm et al., 2016). Additionally, some studies suggest that ecological and evolutionary processes, rather than island biogeography theories, primarily govern species assembly on islands (HilleRisLambers et al., 2012; Mittelbach and Schemske, 2015). Research on microbial communities in seawater indicates that species replacement significantly contributes to β-diversity patterns (Lyu et al., 2023).

This work randomly selected seagrass patches of varying sizes, inculding *Enhalus acoroides* and *Thalassia hemprichi*. Microorganisms attached to the leaves and those present in the surrounding seawater, were systematically sampled. The study analyzed the patterns of β-diversity, considering both taxonomic and functional aspects, and used null models to investigate the mechanisms of microbial community assembly in fragmented seagrass beds. The hypotheses are as follows: (1) The phylogenetic relationships of metapopulation provide a more comprehensive background for community assembly within fragmented seagrass bed habitats. Stochastic processes predominantly shape the constitution of local species pools, thereby influencing on community assembly. (2) Replacement processes are the primary driver of β-diversity differences in microbial communities. (3) Microbial functions play a significant role in community assembly through mechanisms that involve environmental adaptation and niche differentiation. Despite taxonomic differences, functional convergence suggests functional redundancy.

## 2 Materials and methods

### 2.1 Sampling

**Figure 1:**
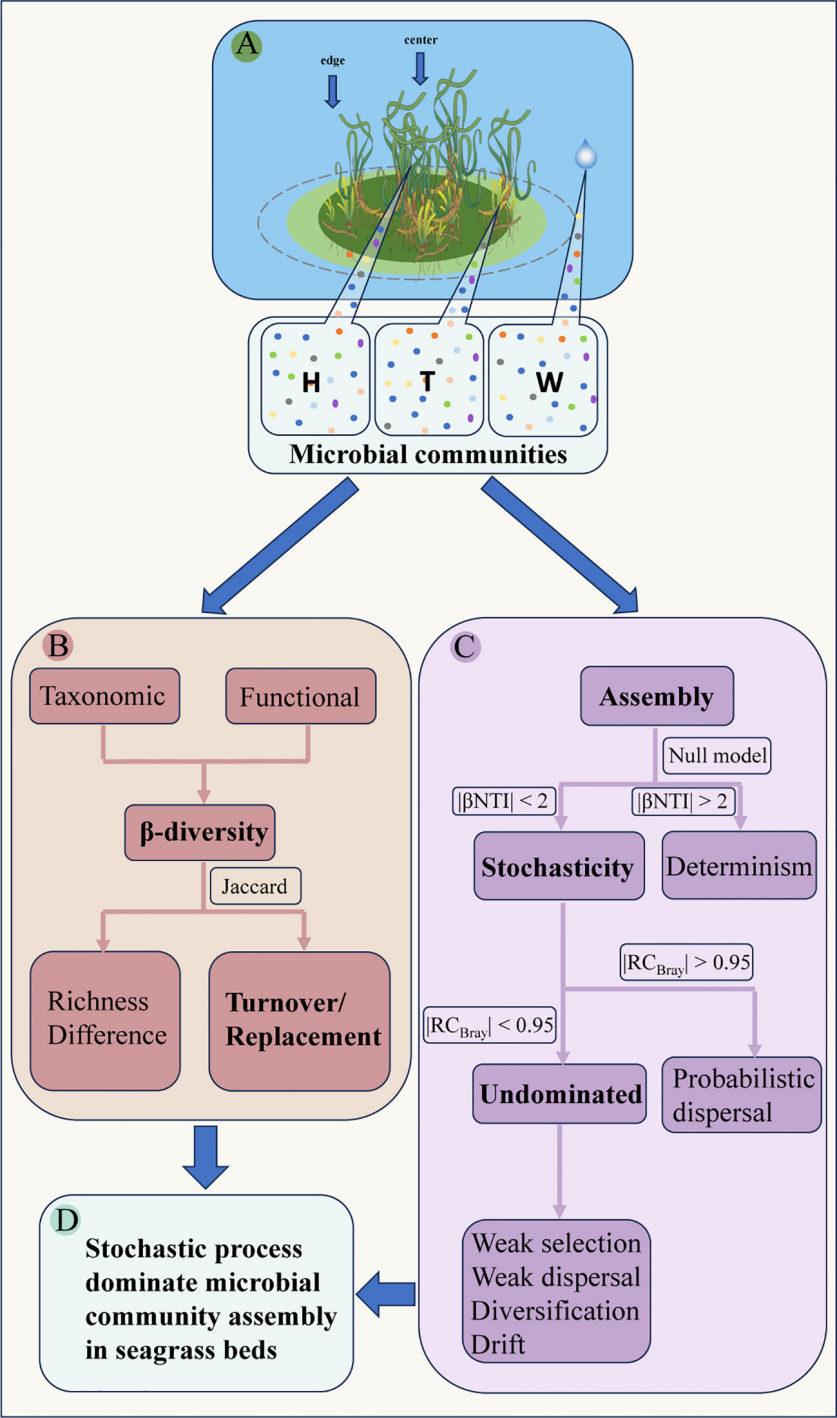
Schematic diagram of microbial community assembly and β-diversity patterns in seagrass beds. (**A**, conceptual diagram of sampling; **B**, anaylsis of β-diversity; **C**, anaylsis of assembly process; **D**, the main conclusion; W, surrounding seawater; H, *E. acoroides*; T, *T. hemprichii*).

Microbial samples were collected in July 2022 from a fragmented seagrass bed area located between Nanhai Village and Baozhi Village (19.4741°N – 19.4784°N, 110.8096°E – 110.8192°E) in Wenchang City, within the South China Sea. A total of 43 seagrass patches were selected, with 27 patches dominated by *Enhalus acoroides*, 13 patches dominated by *Thalassia hemprichii*, and 3 patches co-dominated by both seagrasses. Each patch had designated sampling points at the center and the edge, where microbial samples were collected from seagrass leaf and the surrounding seawater (**Figure 1A**). Specifically, microbial samples from the seawater and seagrass leaves were collected at the center and edge of *E. acoroides* patches (including patches with both seagrasses). For *T. hemprichii* patches, also including 3 patches with both seagrasses, microbial samples were collected from seagrass leaves at the center and edge. In total, we obtained 60 seawater microbial samples, 60 microbial samples attached to *E. acoroides*, and 32 microbial samples attached to *T. hemprichii*, with half at the center and half at the edge of patches. Environmental variables, such as total nitrogen (TN), total phosphorus (TP), and phosphate (PO_4_^3-^), as well as patch characteristics, such as seagrass biomass, patch area, and isolation, were measured. Both environmental variables and patch characteristics are classified as environmental factors.

Microbial samples were filtered onto 0.22 μm diameter filter membranes, with seawater initially filtered through a 5 μm before further filtration to 0.22 μm, with a volume of 1 L. Microorganisms from seagrass leaves were gently brushed into distilled water and subsequently filtered onto the filter membranes. The membranes were stored at -80°C until genomic DNA extraction. Another 1 L seawater sample was collected and transported promptly to the laboratory for measuring TN, TP, and PO_4_^3-^ concentrations, using spectrophotometer and molybdenum blue methods (Murphy and Riley, 1962). Seagrass biomass, including both aboveground and belowground parts, was weighted at wet and dry conditions. Patch morphological assessments and patch areas measurements were conducted using unmanned aerial vehicles (DJI Mini 4 Pro) and GPS markers.

### 2.2 DNA sequencing

Microbial DNA was extracted from the filter membranes using a Thermo genomic DNA extraction kit. A biological company was contracted for high-throughput sequencing services. The OD260/280 values of the DNA samples were within the range of 1.8 to 2.0. The concentration, determined using a Nanodrop 2000 spectrophotometer, exceeded 50 μg/μL, with a total amount surpassing 2 μg. The DNA samples mentioned above were diluted to 1 ng/μL with sterile water and used as a template for PCR amplification of the 16S rRNA target gene. High-fidelity enzymes and primers with barcode tags were used for the amplification. After amplification, PCR products of equal volumes were mixed based on their concentration, and 2% agarose gel electrophoresis was conducted for detection. Subsequently, the target bands were purified using gel extraction kits from QIAGEN. Finally, the raw sequencing data was obtained though sequencing with the Hiseq2500 platform and library preparation using Illumina kits.

The processes of assembly, quality control, and filtering are necessary to convert data into valid data. Subsequently, the Uparse software (Edgar, 2013) was used with a sequence similarity threshold of 97% (Schloss and Handelsman, 2005) to obtain operational taxonomic units (OTUs). After taxonomic annotation based on representative sequences and normalization of OTUs abundance information (Green et al., 2017) based on the minimum sequence count, a total of 4320 OTUs were obtained from seawater, 6739 from *E. acoroides*, and 3157 from *T. hemprichii*, respectively. The taxa, classified at the Family level by OTUs, were used for data analysis in this study. All sequences utilized in this study are publicly accessible at the NCBI Sequence Read Archive (http://www.ncbi.nlm.nih.gov/Traces/sra) under accession PRJNA1048314.

### 2.3 Analysis of microbial community assembly

The microbial community assembly was quantified using a well-accepted null model (Chase et al., 2011) that evaluates the deterministic and stochastic processes based on the abundance of microbial taxonomic groups and their phylogenetic evolutionary distances. This analysis involved 999 randomizations. The phylogenetic relatedness of taxa within the community was evaluated using nearest taxon index (NTI) and its calculation was performed using the “picante” package (Webb et al., 2008). A homogeneous assembly is indicated by NTI values >2 or mean NTI > 0 (at the regional level), while heterogeneous assembly is suggested by NTI values < -2 or mean NTI < 0 (at the regional level) (Kembel, 2009; Stegen et al., 2013).

The β-NTI (Webb et al., 2008; Kembel et al., 2011) and RC_Bray_ (Stegen et al., 2013) values were used to quantify deterministic and stochastic processes. These values were calculated using the “pNST” function in the “NST” package. Assembly of community is considered deterministic if |β-NTI| > 2, while stochastic processes dominate assembly if |β-NTI| < 2 (Kembel et al., 2011). The RC_Bray_ value provides a more precise understanding of stochastic processes. If |RC_Bray_| is greater than 0.95, it is likely that community assembly is dominated by probabilistic dispersal. On the other hand, if |RC_Bray_| is less than 0.95, community assembly can be attributed to undominated processes such as weak selection, weak dispersal, diversification, and drift (Stegen et al., 2015) (**Figure 1C**). Stegen et al. (2013) developed a statistical framework to describe the ecological processes of community assembly (**Figure 1C**).

To explore the impact of environmental factors on community assembly, the mantel test based on matrix data was employed to observe the correlation between the two. Euclidean distances for both environmental variables and patch characteristics were calculated using the “dist” function of the “vegan” R package, resulting in matrix data. The significance threshold was set at *p* < 0.05. The patch isolation was calculated based on the coordinates between sampling sites using the “distm” function in the “geosphere” R package.

### 2.4 Analysis of microbial community β-diversity patterns

A β-diversity partitioning approach based on the Jaccard dissimilarity index (Podani and Schmera, 2011; Carvalho et al., 2012; Cardoso et al., 2014) was used to calculate three paired β-diversity metrics for both microbial taxonomy and ecological functional groups (referred to as “functional”): total diversity (Total), turnover or replacement (Repl), and richness differences (RichDiff) (**Figure 1B**). Ternary plots were utilized to illustrate the varying contributions of different metrics to β-diversity differences. The differences between taxonomic and functional were compared for each diversity metric, and their correlation was explored using the mantel test. Additionally, the mantel test was used to indicate the relationship between the β-diversity metrics and environmental factors.

The β-diversity analysis in microbial taxonomy employed the data obtained from the classification of OTUs at the Family level, which yielded 416 taxa in seawater, 464 taxa in *E. acoroides*, and 364 taxa in *T. hemprichii*. Ecological function groups of microbial communities in seagrass beds were obtained by comparing OTUs classification tables with the functional annotation of prokaryotic taxa (FAPROTAX) (http://www.zoology.ubc.ca/louca/FAPROTAX/) using Python scripts. These functional groups effectively explained and predicted the functions related to the cycling of elements such as carbon (C), nitrogen (N), phosphorus (P), and sulfur (S) (Louca et al., 2016; Kumar et al., 2018; Picazo et al., 2021). A total of 57 ecological functional groups were identified from microbial communities of seawater and *E. acoroides*, and 48 in *T. hemprichii*. These data were used for β-diversity analysis in microbial functionality.

The analyses were conducted using the R software (Team, 2023). The β-diversity calculation used the “adespatial” package, the mantel test used the “vegan” package, and functional annotations were based on the Linux system and accomplished using the “FAPROTAX” package.

## 3 Results

### 3.1 Stochasticity, undominated process specifically, determines microbial community assembly

**Figure 2:**
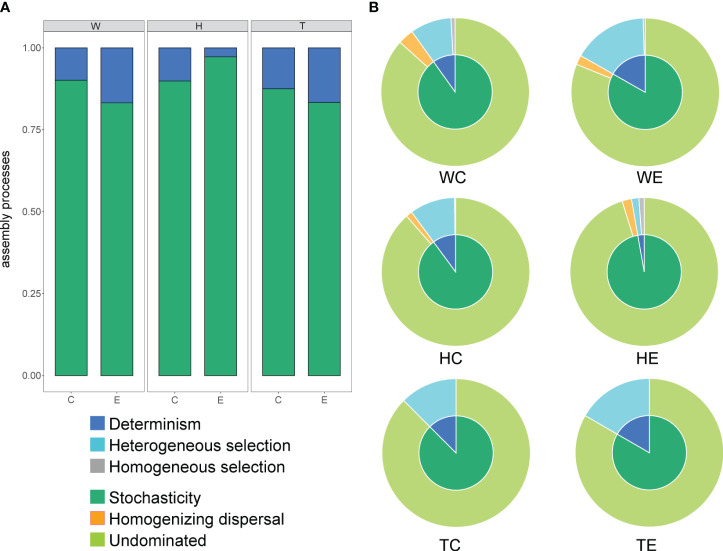
The proportion of different assembly processes of microbial communities in seagrass beds (**A**, the proportion of deterministic and stochastic processes; **B**, the proportion of components of deterministic and stochastic processes; W, surrounding seawater; H, *E. acoroides*; T, *T. hemprichii*; C, center of the patch; E, edge of the patch).

The average NTI values of microbial communities in both seagrass species are higher than those in the surrounding seawater, and both are greater than zero (**Supplementary Figure S1**). This suggests a homogeneous assembly of communities in seagrass beds and a closer phylogenetic relationship in the microbial communities attached to seagrasses. The assembly of microbial communities in seagrass beds is primarily governed by stochastic processes, accounting for over 75%, as evidenced by β-NTI values (**Supplementary Figure S2**; **Figure 2A**). The contribution of deterministic processes in seawater and *T. hemprichii* patches is higher at the edge than at the center, while the opposite is observed in *E. acoroides* patches (**Figure 2A**). Further identification revealed that determinism is primarily attributed to heterogeneous selection, whereas stochasticity is mainly attributed to undominated processes, including weak selection, weak dispersal, diversification, and drift (**Figure 2B**).

Mantel test results (**Supplementary Table S1**) indicate a significant correlation between the assembly processes of microbial communities (β-NTI) at the centers (*p* < 0.05) of *E. acoroides* patches with seagrass biomass and a partially significant correlation (*p* < 0.08) at the edges. At the centers of *T. hemprichii* patches, community assembly is partially correlated with TN and isolation, while at the edges, it is significantly correlated with PO_4_^3-^ (**Supplementary Table S1**). We analyzed whether there were patterns of variation in dominant taxa, defined as taxa with abundances exceeding 1% across all sampling sites, within the community in fragmented seagrass bed patches (**Supplementary Figure S3**). The results showed no discernible patterns, suggesting that dominant taxa do not dictate the assembly of microbial communities in fragmented seagrass beds.

### 3.2 Replacement is responsible for the β-diversity difference of microbial communities

**Figure 3:**
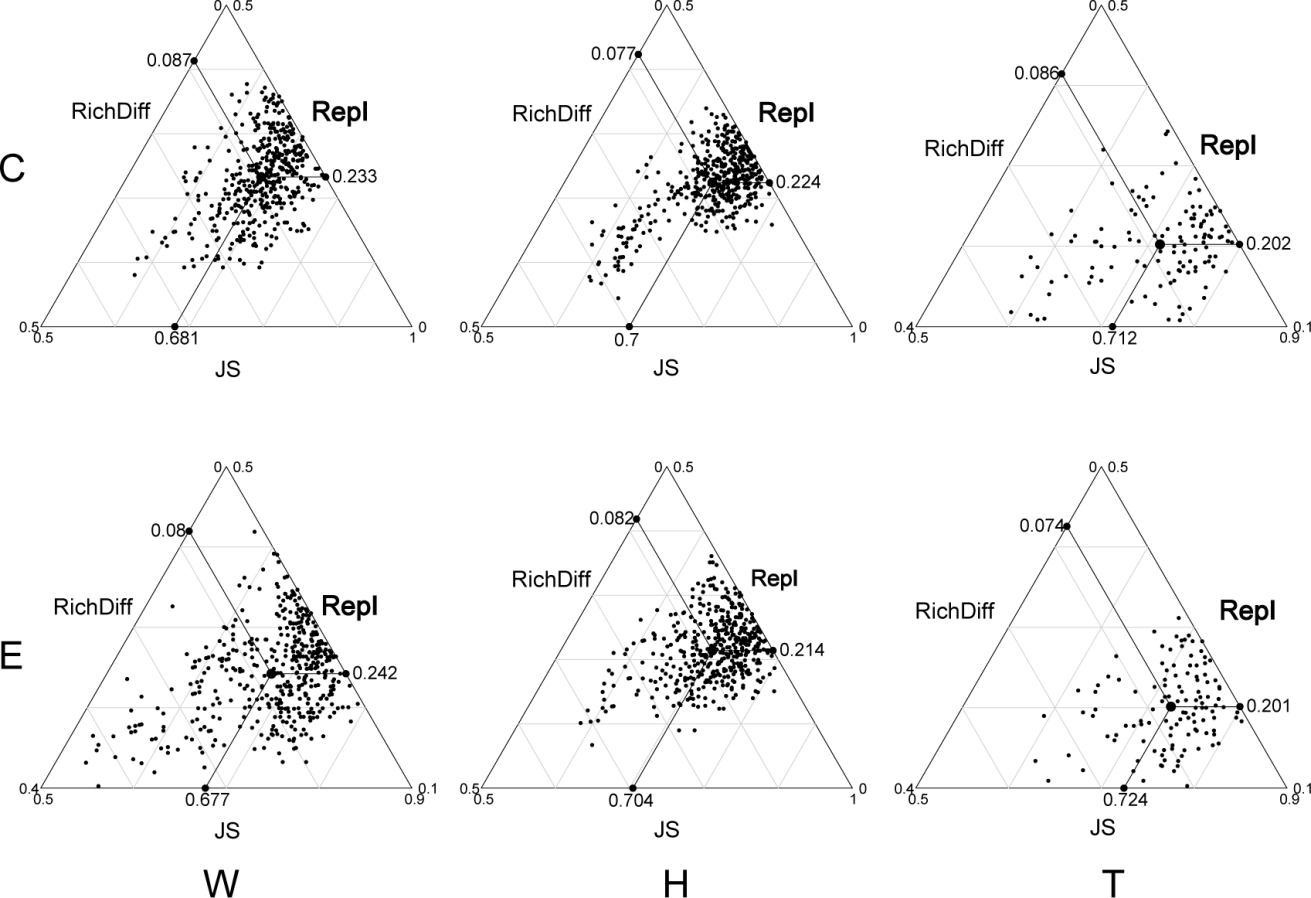
Triangular plots of taxonomic β-diversity comparisons (using Jaccard dissimilarity index) in seagrass beds among all sites (Its position is determined by a triplet of values from the JS (Jaccard Similarity), Repl (replacement) and RichDiff (richness difference) matrices. The mean values of JS, Repl and RichDiff are shown. Each point represents a pair of sites with 435 pairs sites in the W and H, 120 pairs sites in the T. W, surrounding seawater; H, *E. acoroides*; T, *T. hemprichii*; C, center of the patch; E, edge of the patch.).

The analysis of β-diversity partition indicates that the differences in microbial community composition are primarily driven by species replacement, which contributes significantly more to β-diversity than richness differences (**Figure 3**; **Table 1**). Furthermore, no significant differences were observed between seawater and the two seagrass species, nor between the center and edge of patches. In seawater microbial communities, species replacement processes accounted for an average of 73.96%, while richness differences processes accounted for 26.04% (**Table 1**). In the microbial communities attached to *E. acoroides*, species replacement processes were 73.42% and richness differences processes were 26.58%. For the microbial communities attached to *T. hemprichii*, species replacement processes accounted for 71.65% and richness differences processes accounted for 28.35% (**Table 1**). The mantel test revealed that the species replacement processes of most communities did not exhibit significant correlation with environmental factors (**Supplementary Table S2**). Specifically, the replacement process was significantly and positively correlated with patch area, isolation, and PO_4_^3-^ in the central community of *E. acoroides* patches. TP and TN were significantly and positively correlated with the replacement process at the edge of *T. hemprichii* patches (**Supplementary Table S2**).

**Table 1:** The contribution of replacement and richness differences to β-diversity differences (W, surrounding seawater; H, *E. acoroides*; T, *T. hemprichii*; C, center of the patch; E, edge of the patch).

		W-C	W-E	H-C	H-E	T-C	T-E
Taxonomy	Replacement	0.7281	0.7511	0.7446	0.7238	0.7025	0.7304
	Richness difference	0.2719	0.2489	0.2554	0.2762	0.2975	0.2696
Function	Replacement	0.5742	0.5111	0.4108	0.4585	0.4269	0.3121
	Richness difference	0.4258	0.4889	0.5892	0.5415	0.5731	0.6879

**Figure 4:**
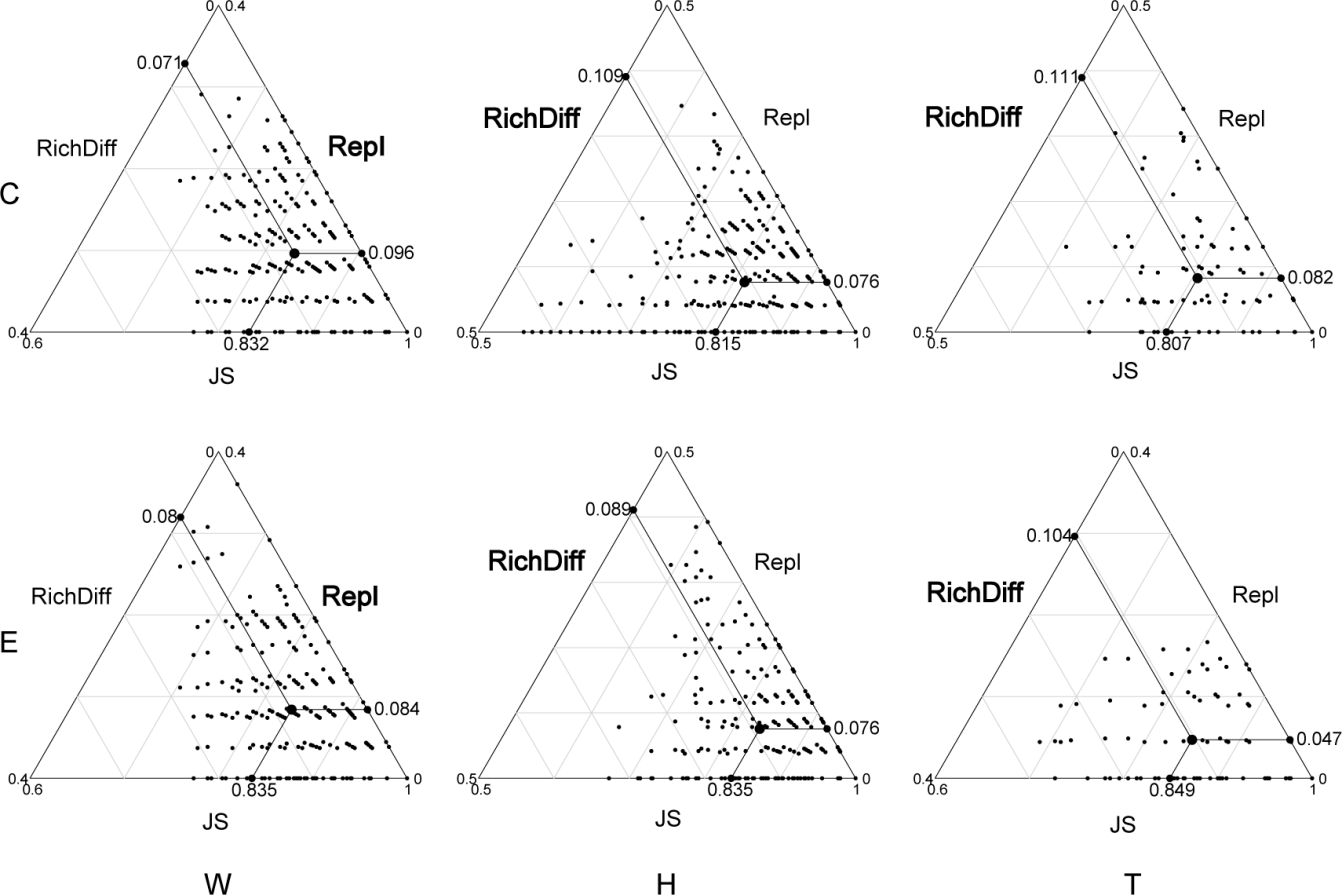
Triangular plots of functional β-diversity comparisons (using Jaccard dissimilarity index) for microbes in seagrass beds among all sites. (Its position is determined by a triplet of values from the JS (Jaccard Similarity), Repl (replacement) and RichDiff (richness difference) matrices. The mean values of JS, Repl and RichDiff are shown. Each point represents a pair of sites with 435 pairs sites in the W and H, 120 pairs sites in the T. W, surrounding seawater; H, *E. acoroides*; T, *T. hemprichii*; C, center of the patch; E, edge of the patch.).

Among the top 15 most abundant ecological function groups, the majority are associated with the C cycle (**Supplementary Figure S4**). However, their relative abundance varies. Chemoheterotrophy and aerobic_chemoheterotrophy are most abundant in the microbial communities of seawater and *T. hemprichii*, whereas the opposite is true for *E. acoroides* (**Supplementary Figure S4**). In contrast to differences in community composition, microbial functional differences in seawater are mainly contributed by replacement processes, whereas in seagrass richness differences play a predominant role (**Figure 4**; **Table 1**). Specifically (**Table 1**), in seawater microbial communities, replacement processes contribute an average of 54.27%, while richness difference processes contribute 45.73%. For the communities attached to *E. acoroides*, replacement processes contribute an average of 43.47% and richness differences processes contribute 56.53%. In the microbial communities attached to *T. hemprichii*, replacement processes were 37%, and richness differences processes contributed 63%. Replacement processes of most communities were not significantly correlated with environmental factors, except for a significant positive correlation with PO_4_^3-^ and patch isolation in seawater at the edge of patches (**Supplementary Table S3**). There was no significant correlation between all richness difference processes and environmental factors (**Supplementary Table S4**).

**Figure 5:**
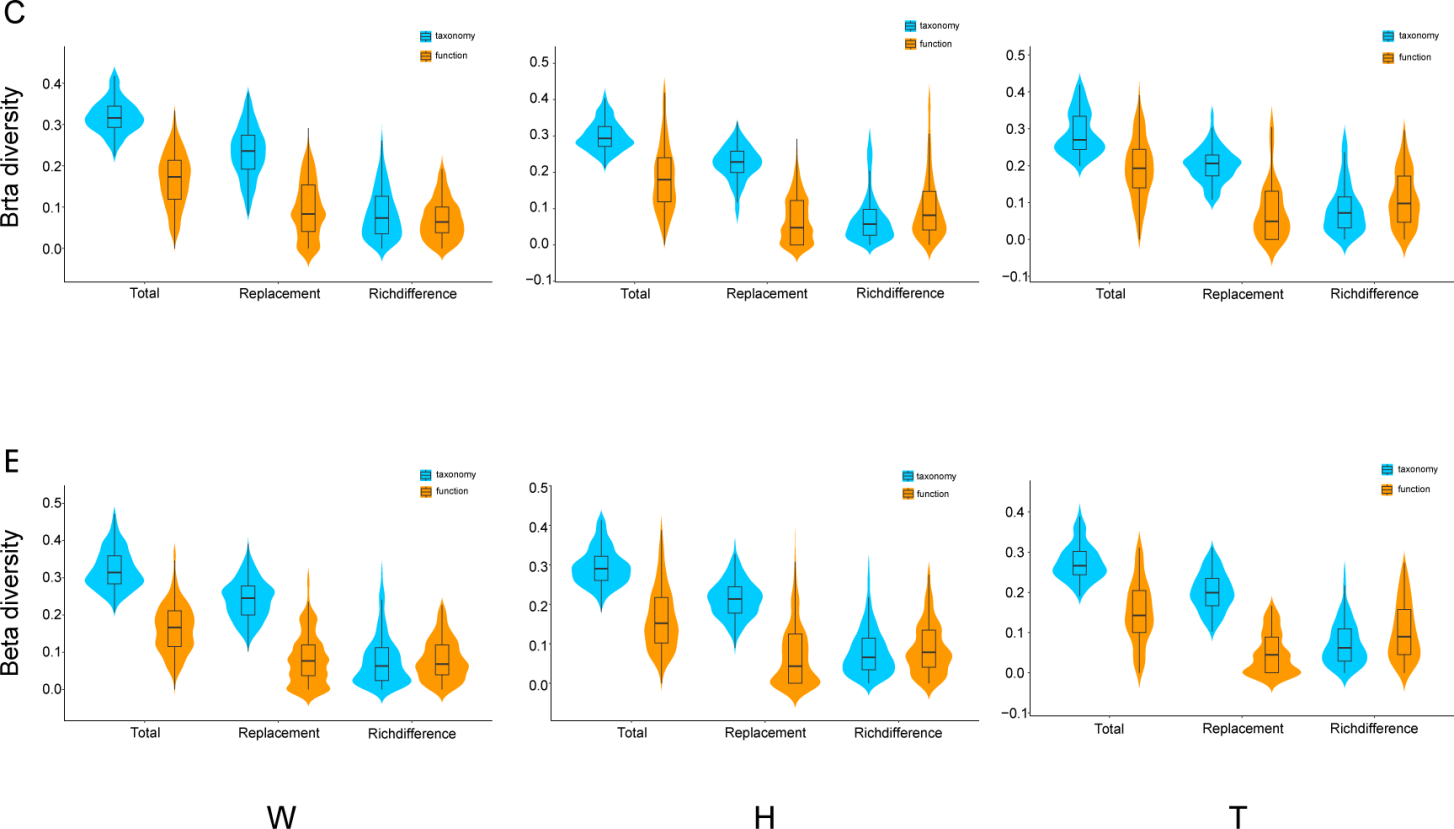
Comparisons of β-diversity components (total, replacement and richness difference) for taxonomy and ecological function groups of microorganisms in seagrass beds (W, surrounding seawater; H, *E. acoroides*; T, *T. hemprichii*; C, center of the patch; E, edge of the patch.).

A comparison was conducted between taxonomy and functionality for the three components of β-diversity: total β-diversity, replacement, and richness differences. The results indicate that both total β-diversity and replacement exhibit higher values in taxonomy than in functionality, while richness differences are relatively similar between the two (**Figure 5**). Mantel analyses were performed to examine the relationship between taxonomic and functional β-diversity. Significant positive correlations were found for total β-diversity in seawater, including both the center and edge. Only at the edge of *E. acoroides* patches, total β-diversity exhibited a significant positive correlation. At the center and edge of *T. hemprichii* patches, significant positive correlations of total β-diversity and richness difference were observed. However, replacement processes existed a partially significant positive correlation at the center (**Table 2**).

**Table 2:** Mantel test between the β-diversity components (total, replacement and richness difference) and taxonomy, ecological function groups of microorganisms in seagrass beds (Bold numbers indicates significant correlation, *p* < 0.05.

	W-C		W-E		H-C		H-E		T-C		T-E	
	r	*p*	r	*p*	r	*p*	r	*p*	r	*p*	r	*p*
Total	0.3975	**1e-04**	0.3654	**1e-04**	0.0393	0.3594	0.2873	**0.0165**	0.4033	**0.0171**	0.4873	**0.0053**
Replacement	0.1261	0.1319	0.0827	0.2113	-0.1672	0.9596	0.0154	0.4349	0.1997	0.062	0.0129	0.4781
Richness Difference	-0.0455	0.7204	0.0423	0.2709	-0.0252	0.5471	0.0662	0.1983	0.3285	**0.0085**	0.3097	**0.0183**

W, surrounding seawater; H, *E. acoroides*; T, *T. hemprichii*; C, center of the patch; E, edge of the patch).

## 4 Discussion

While it is widely recognized that both deterministic and stochastic processes impact community assembly (Ofiţeru et al., 2010; Chase and Myers, 2011; Zhou and Ning, 2017), there is an ongoing debate regarding their relative importance in governing community variations (Zhou et al., 2013; Vellend et al., 2014). Notably, the significance of stochastic processes has historically been understated in this discourse (Zhou and Ning, 2017; Li and Gao, 2023). The study highlights the significant role of stochastic processes in microbial community assembly in fragmented seagrass beds, in contrast to traditional island ecology perspectives. Additionally, the study reveals differences in the patterns of functional and taxonomic β-diversity, including the relative contributions of replacement and richness difference processes and their responses to environmental factors. This aids in a better understanding and elucidation of these stochastic rules. On the basis of the dominant factor of stochastic assembly, perhaps focusing on community function in future management and restoration is an ingenious perspective.

### 4.1 The stochastic assembly processes in microbial communities

The NTI results reflect the phylogenetic clustering relationships within communities. An average NTI across multiple communities at the regional level demonstrates the homogeneous assembly of microbial communities across the region, rather than relying on a single NTI. In other words, the phylogenetic relationships of metapopulation provide background information for the microbial community assembly in seagrass beds. According to Stegen et al. (2013), undominant processes in stochasticity, such as weak selection, weak dispersal, diversification, and drift, are responsible for the assembly of microbial communities. The mantel results indicate a lack of significant correlation between β-NTI and environmental factors, supporting this notion. The findings suggest that community assembly in seagrass bed patches is minimally explained by environmental variables and patch characteristics, which is consistent with previous research on microbes (Ramette and Tiedje, 2007; Zhou et al., 2008; Graham et al., 2016).

Generally, diversification and dispersal are often considered components of stochastic processes, especially in microbial communities (Chase and Myers, 2011), as microbial dispersal is typically passive (Nemergut et al., 2013) and passive dispersal is regarded as random (Vellend et al., 2014). Drift refers to the random changes in the relative abundance of different species within a community over time, driven by inherent stochastic processes such as birth and death (Vellend, 2010; Nemergut et al., 2013). It is undeniably stochastic (Vellend et al., 2014). The challenge lies in the inability to directly measure these stochastic processes in community ecology. Furthermore, in microbial communities, drift is likely associated with functional redundancy and plays a crucial role in shaping community structure (Ofiţeru et al., 2010; Zhou et al., 2013), as the common functional redundancy in microbial communities can induce drift by increasing neutrality (Allison and Martiny, 2008; Delgado-Baquerizo et al., 2016). It is clear that there are dynamic exchanges between microorganisms attached to seagrass and the species pool in the surrounding seawater (Fahimipour et al., 2017). Seagrasses prevent or selectively filter microbial colonization for various reasons. Combining our results, it can be inferred that stochastic processes have a significant impact on the assembly of attached microbial communities by predominantly shaping the formation of local species pools.

### 4.2 Microbial community assembly from a β-diversity patterns perspective

Understanding the contributions of the two components of β-diversity is crucial for comprehending how communities change, and provides a new perspective on community assembly (Carvalho et al., 2012). From a taxonomic standpoint, differences in microbial community β-diversity are mainly driven by species replacement. From a functional perspective, replacement processes contribute slightly more than richness differences in seawater microbial communities, which is contrary to the observed pattern in communities attached to seagrasses. These findings align with related research on seawater microbial communities (Sun et al., 2024). Furthermore, we observed that the proportion of dominant taxa did not exhibit consistent patterns with increasing or decreasing patch area of seagrass beds. This statement contrasts with the area-diversity theory in island ecology, which suggests that species diversity is proportional to island size (Li et al., 2020). The area-diversity theory is not applicable to fragmented seagrass bed patches; instead, replacement processes dominate the β-diversity differences in microbial communities. Previous studies suggest that species dispersal may cause species turnover among communities (Swenson et al., 2011). Dispersal is common in microbial communities (Wu et al., 2021).

Taxonomically, β-diversity is primarily influenced by replacement processes, while the contribution of richness differences to functional β-diversity slightly surpasses that of replacement. This implies a significant convergence of functional traits among communities (Carvalho et al., 2020), reinforcing the concept of heightened functional redundancy within microbial communities. The taxonomic dissimilarity and functional convergence suggest turnover among microbial taxa with similar functions (Wu et al., 2021). There is a weak yet significant correlation between taxonomic and function-based β-diversity components, indicating that these two facets provide complementary ecological information (Devictor et al., 2010). This pattern has been observed across diverse biological groups, including fish, planktonic plants, and zooplankton, as well as various habitats, encompassing different types of water bodies (Gianuca et al., 2018; Wu et al., 2018; Hill et al., 2019). Furthermore, there is limited correlation between β-diversity components from both taxonomic and functional perspectives and environmental factors, including environmental variables and patch characteristics. These findings suggest that environmental factors play a minimal role in driving replacement processes within microbial communities. The collective findings demonstrate that the β-diversity patterns provide a new perspective and reference for better understanding the stochastic assembly rules of microbial communities in fragmented seagrass beds.

Research indicates a complex and intimate interaction between plants and their associated microorganisms, whether in the rhizosphere microbiome (Gabriel Nuto et al., 2023; Tang et al., 2023) or the phyllosphere microbiome (Lehnen et al., 2016; Ettinger et al., 2017). Seagrasses are no exception, as changes in the structure and diversity of attached microbial communities, along with their responses to the environment, significantly impact the health, growth, and functions of seagrasses (Chen et al., 2022; O’Connor et al., 2022). For instance, alterations in rhizosphere microbial communities play a pivotal role in the successful transplantation of seagrasses (Christiaen et al., 2013). Undoubtedly, the ecosystem services provided by seagrass beds, such as high productivity and a robust carbon sequestration capacity (Fourqurean et al., 2012; Han et al., 2023), predominantly rely on seagrasses. Therefore, delving into community assembly mechanisms is crucial for understanding of the intricate relationship between microbial communities and seagrasses. This knowledge can guide the development of targeted management strategies to protect and restore seagrass bed ecosystems.

## 5 Conclusion

The assembly processes of microbial communities attached to seagrass play a crucial role in the growth and health of seagrasses and, consequently, the stability, diversity, and multifunctionality of seagrass bed ecosystems. We emphasize the dominance of stochastic rules, particularly undominant processes such as weak selection, weak dispersal, diversification, and drift, in the assembly of these communities (**Figure 1D**). The taxonomic-based differences in β-diversity are driven by replacement processes, whereas in functional β-diversity, the contribution of richness differences slightly surpasses that of replacement processes. Although there are significant taxonomic distinctions among microbial communities in seagrass beds, functional convergence and redundancy are observed. Environmental factors, indicative of deterministic processes, provide minimal explanation for the patterns of β-diversity. In summary, stochastic processes govern the assembly of microbial communities in fragmented seagrass beds. The taxonomic and functional β-diversity patterns provide a new perspective and support for better comprehending these stochastic assembly rules. This study provides foundational evidence for the mechanisms of microbial community assembly in fragmented seagrass beds, offering novel insights for the conservation and management of seagrass beds.

## Data availability statement

The datasets presented in this study can be found in online repositories. The names of the repository/repositories and accession number(s) can be found in the article/**Supplementary Material**.

## Ethics statement

The manuscript presents research on animals that do not require ethical approval for their study.

## Author contributions

XN: Conceptualization, Formal analysis, Writing – original draft, Visualization. WR: Formal analysis, Methodology, Writing – review & editing. CX: Data curation, Methodology, Writing – review & editing. RW: Data curation, Methodology, Writing – review & editing. JZ: Formal analysis, Methodology, Writing – review & editing. HW: Conceptualization, Data curation, Funding acquisition, Methodology, Project administration, Supervision, Writing – review & editing.
