# Corrigendum: Taxonomic and functional β-diversity patterns reveal stochastic assembly rules in microbial communities of seagrass beds

**DOI:** 10.3389/fpls.2024.1470123

**Published:** 2024-08-06

**Authors:** Xiaofeng Niu, Wenjing Ren, Congjun Xu, Ruilong Wang, Jingwei Zhang, Huan Wang

**Affiliations:** ^1^ School of Marine Biology and Fisheries, State Key Laboratory of Marine Resource Utilization in South China Sea, Hainan University, Haikou, Hainan, China; ^2^ Institute of Hydrobiology, Chinese Academy of Sciences, Wuhan, Hubei, China

**Keywords:** stochastic assembly, β-diversity, replacement, attached microbial communities, seagrass bed, habitat fragmentation

In the published article, there was an error in the Funding statement. For the Hainan University Start-up Funding for Scientific Research, “KYOD (ZR)-23, 086” and “KYOD (ZR)-23, 087” were incorrectly used instead of “KYQD (ZR)-23, 086” and “KYQD (ZR)-23, 087”. The correct Funding statement appears below.

**FUNDING**

The author(s) declare financial support was received for the research, authorship, and/or publication of this article. This research was supported by the National Natural Science Foundation of China (Grant No. 32001151 and 42377469) and Hainan University Start-up Funding for Scientific Research (KYQD (ZR)-23, 086) and Hainan University Start-up Funding for Scientific Research (KYQD (ZR)-23, 087).

The authors apologize for this error and state that this does not change the scientific conclusions of the article in any way. The original article has been updated.

