# Supplementary material for: Taxonomic and functional β-diversity patterns reveal stochastic assembly rules in microbial communities of seagrass beds
Source: Front Plant Sci. 2024 Feb 28;15:1367773. doi: 10.3389/fpls.2024.1367773 (PMC10932972; doi:10.3389/fpls.2024.1367773)
Supplement: Supplementary file 1 [file DataSheet_1.docx]

**Taxonomic and functional β-diversity patterns reveal stochastic assembly rules in microbial communities of seagrass beds**

Xiaofeng Niu^1,2^, Wenjing Ren^1^, Congjun Xu^2^, Ruilong Wang^2^, Jingwei Zhang^2^, Huan Wang^1,2*^

*^1^School of Marine Biology and Fisheries, State Key Laboratory of Marine Resource Utilization in South China Sea, Hainan University, Haikou, Hainan 570228, P. R. China*

*^2^Institute of Hydrobiology, Chinese Academy of Sciences, Wuhan 430072, P. R. China*

**^*^Email: wanghuan@hainanu.edu.cn**

**Supplementary Materials**


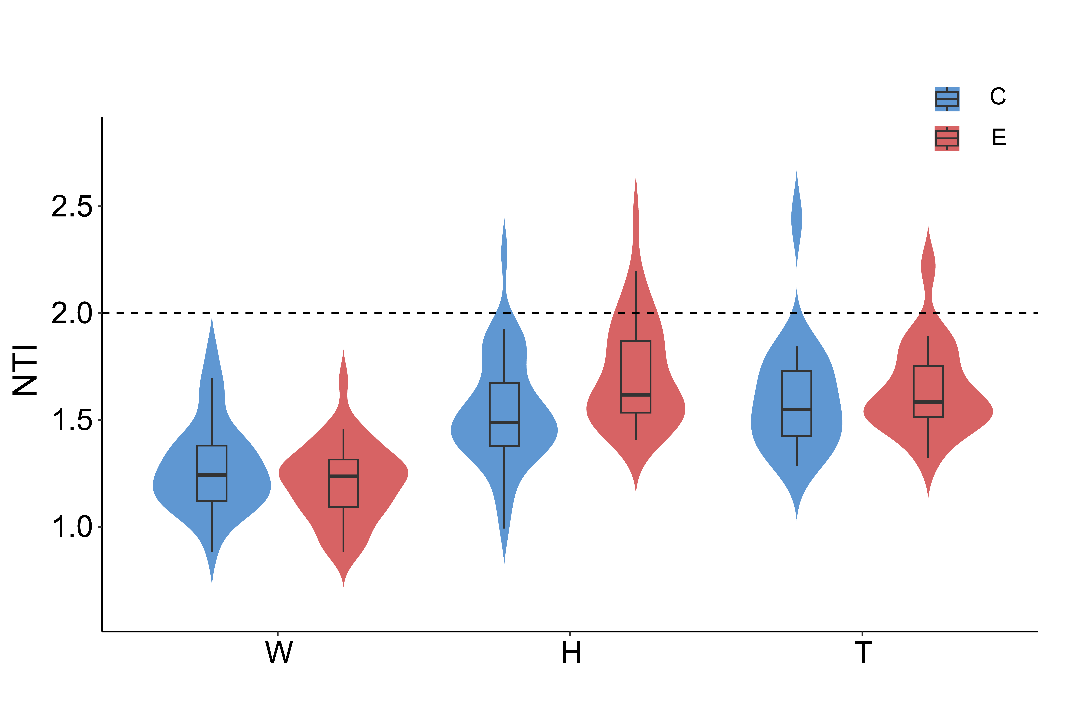


Figure S1 The homogeneous and heterogeneous assembly of microbial communities in seagrass beds (W: surrounding seawater; H: *E. acoroides*; T: *T. hemprichii*; C: center of the patch; E: edge of the patch.)


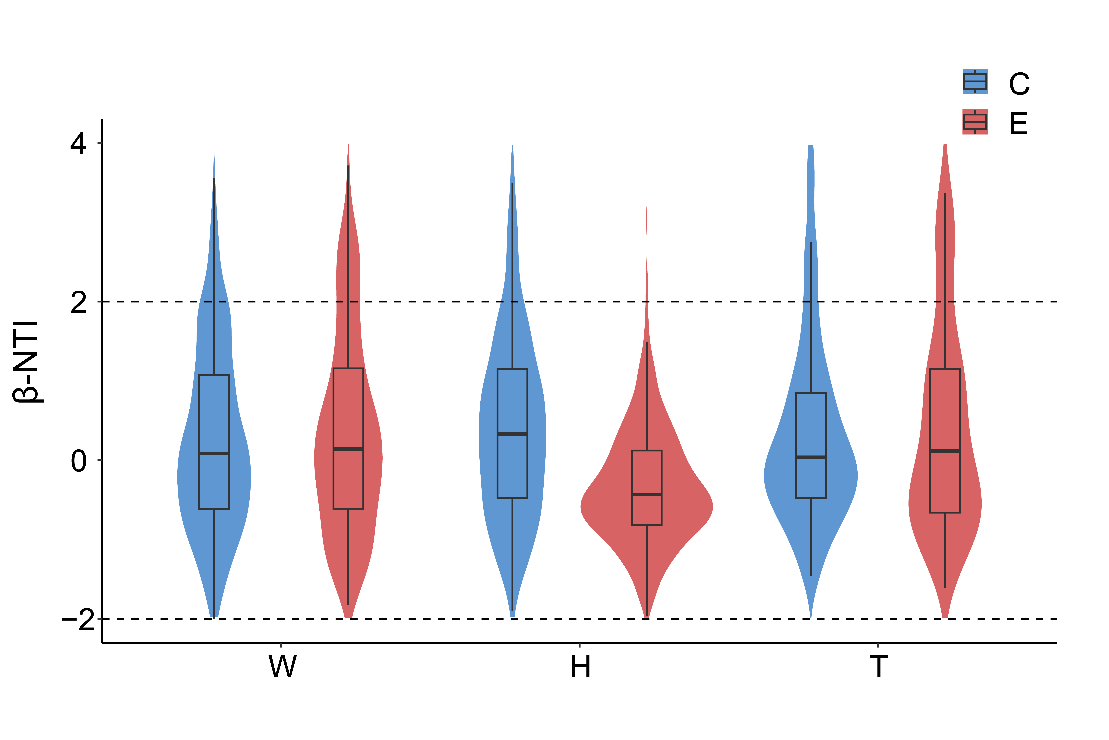


Figure S2 The deterministic and stochastic processes of microbial community assembly in seagrass beds (W: surrounding seawater; H: *E. acoroides*; T: *T. hemprichii*; C: center of the patch; E: edge of the patch.)

Table S1 Mantel test between the β-NTI and environmental factors (Bold numbers indicates significant correlation, *p* < 0.05. W: surrounding seawater; H: *E. acoroides*; T: *T. hemprichii*; C: center of the patch; E: edge of the patch.)

|  | W-C | | W-E | | H-C | | H-E | | T-C | | T-E | |
| --- | --- | --- | --- | --- | --- | --- | --- | --- | --- | --- | --- | --- |
|  | r | *p* | r | *p* | r | *p* | r | *p* | r | *p* | r | *p* |
| TP | -0.04 | 0.6954 | -0.06 | 0.737 | 0.0108 | 0.4573 | -0.089 | 0.8169 | 0.064 | 0.2731 | 0.1419 | 0.1461 |
| TN | -0.0015 | 0.5717 | 0.0109 | 0.4583 | -0.0107 | 0.5364 | 0.0071 | 0.4593 | 0.154 | 0.0508 | 0.036 | 0.3978 |
| acreage | 0.0326 | 0.3295 | -0.1739 | 0.9582 | -0.177 | 0.9589 | -0.003 | 0.504 | 0.099 | 0.1483 | 0.1195 | 0.1574 |
| PO_4_^3-^ | -0.0490 | 0.7781 | -0.2261 | 0.9926 | 0.050 | 0.28 | 0.091 | 0.1656 | -0.056 | 0.7218 | 0.2719 | **0.0187** |
| biomass | - | - | - | - | 0.1968 | **0.0204** | 0.1178 | 0.0863 | 0.002 | 0.4931 | -0.044 | 0.6572 |
| isolation | -0.1992 | 0.9993 | -0.1764 | 0.9975 | -0.003 | 0.5074 | -0.018 | 0.6025 | 0.1383 | 0.074 | -0.057 | 0.6792 |


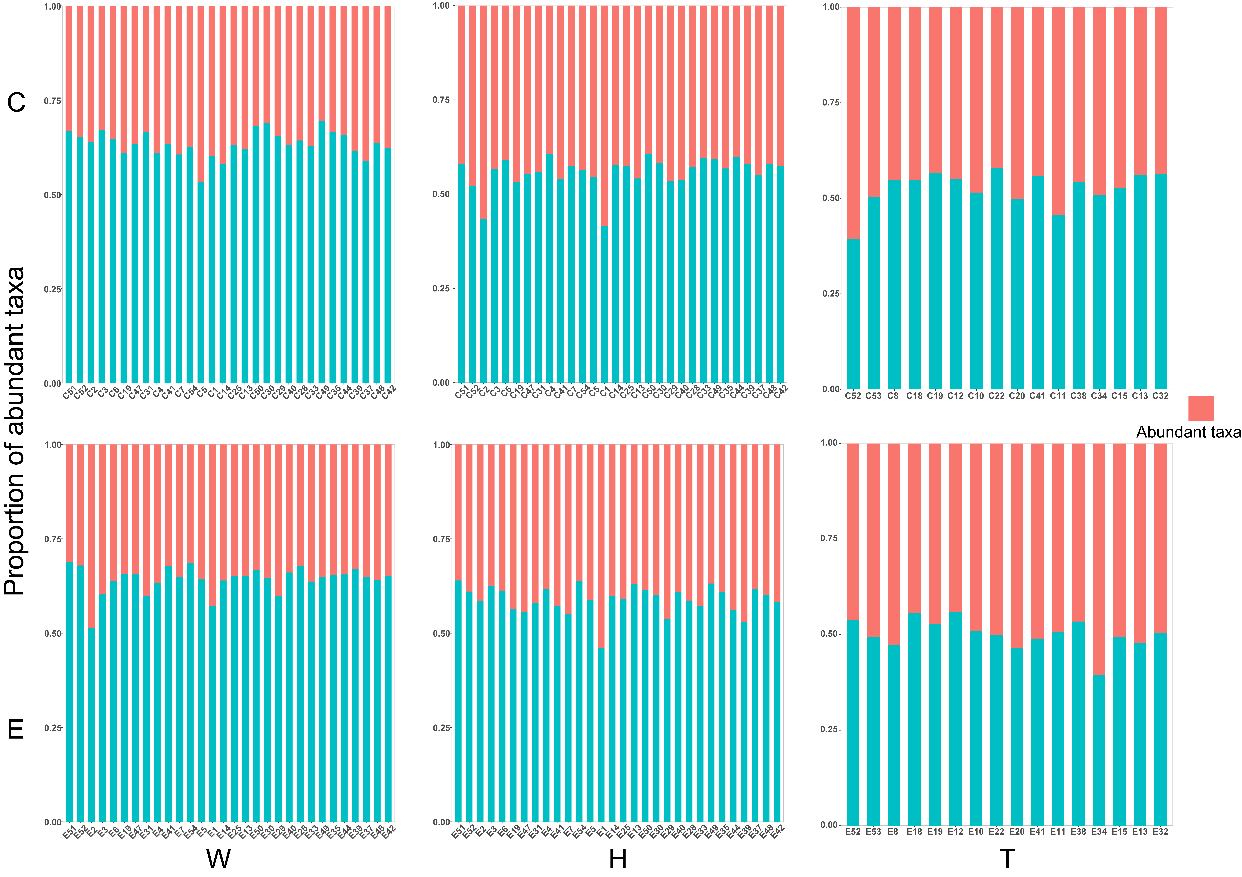


Figure S3 The proportion of abundant taxa in microbial communities of seagrass beds (The sites on the horizontal axis are arranged according to the decreasing patch area. W: surrounding seawater; H: *E. acoroides*; T: *T. hemprichii*; C: center of the patch; E: edge of the patch.)

Table S2 Mantel test between the replacement of taxonomic β-diversity and environmental factors (Bold numbers indicates significant correlation, *p* < 0.05. W: surrounding seawater; H: *E. acoroides*; T: *T. hemprichii*; C: center of the patch; E: edge of the patch.)

|  | W-C | | W-E | | H-C | | H-E | | T-C | | T-E | |
| --- | --- | --- | --- | --- | --- | --- | --- | --- | --- | --- | --- | --- |
|  | r | *p* | r | *p* | r | *p* | r | *p* | r | *p* | r | *p* |
| TP | -0.279 | 0.985 | -0.138 | 0.888 | -0.199 | 0.965 | 0.021 | 0.426 | 0.014 | 0.452 | 0.259 | **0.031** |
| TN | -0.013 | 0.578 | 0.165 | 0.055 | 0.052 | 0.323 | -0.002 | 0.526 | -0.166 | 0.898 | 0.265 | **0.015** |
| acreage | 0.080 | 0.252 | -0.023 | 0.603 | 0.169 | **0.027** | -0.001 | 0.525 | -0.097 | 0.763 | 0.111 | 0.228 |
| PO_4_^3-^ | 0.018 | 0.458 | -0.020 | 0.594 | 0.145 | **0.020** | 0.022 | 0.410 | 0.056 | 0.305 | 0.141 | 0.204 |
| biomass | - | - | - | - | -0.015 | 0.602 | 0.022 | 0.417 | 0.046 | 0.322 | 0.027 | 0.429 |
| isolation | -0.019 | 0.627 | 0.129 | **0.022** | 0.140 | **0.010** | 0.031 | 0.330 | -0.163 | 0.893 | 0.058 | 0.388 |


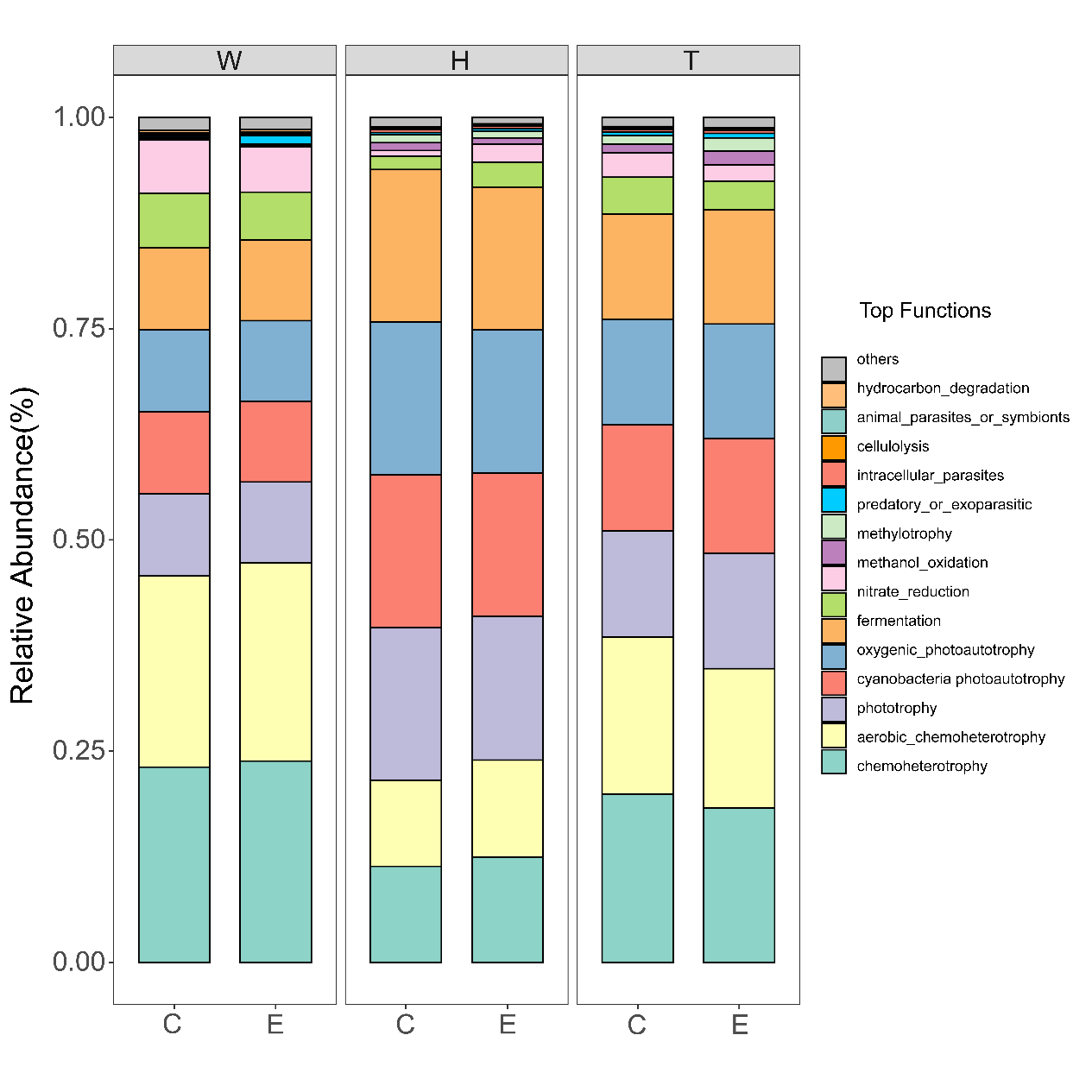


Figure S4 The relative abundance of ecological function groups from microorganism in seagrass beds (Top fifteen abundant taxa were shown. W: surrounding seawater; H: *E. acoroides*; T: *T. hemprichii*; C: center of the patch; E: edge of the patch.)

Table S3 Mantel test between the replacement of functional β-diversity and environmental factors (Bold numbers indicates significant correlation, *p* < 0.05. W: surrounding seawater; H: *E. acoroides*; T: *T. hemprichii*; C: center of the patch; E: edge of the patch.)

|  | W-C | | W-E | | H-C | | H-E | | T-C | | T-E | |
| --- | --- | --- | --- | --- | --- | --- | --- | --- | --- | --- | --- | --- |
|  | r | *p* | r | *p* | r | *p* | r | *p* | r | *p* | r | *p* |
| TP | 0.185 | 0.059 | 0.099 | 0.163 | 0.106 | 0.172 | 0.099 | 0.186 | -0.059 | 0.659 | 0.117 | 0.185 |
| TN | -0.072 | 0.727 | 0.116 | 0.108 | 0.105 | 0.170 | -0.180 | 0.965 | 0.066 | 0.309 | 0.134 | 0.148 |
| acreage | 0.103 | 0.190 | 0.081 | 0.228 | 0.018 | 0.438 | -0.001 | 0.491 | -0.091 | 0.745 | 0.022 | 0.404 |
| PO_4_^3-^ | -0.135 | 0.923 | 0.181 | **0.026** | -0.050 | 0.707 | -0.001 | 0.499 | -0.003 | 0.504 | -0.053 | 0.620 |
| biomass | - | - | - | - | 0.035 | 0.355 | 0.072 | 0.215 | -0.044 | 0.657 | -0.231 | 0.991 |
| isolation | -0.022 | 0.618 | 0.163 | **0.006** | 0.089 | 0.102 | 0.040 | 0.281 | -0.049 | 0.632 | 0.065 | 0.291 |

Table S4 Mantel test between the rich difference of functional β-diversity and environmental factors (Bold numbers indicates significant correlation, *p* < 0.05. W: surrounding seawater; H: *E. acoroides*; T: *T. hemprichii*; C: center of the patch; E: edge of the patch.)

|  | W-C | | W-E | | H-C | | H-E | | T-C | | T-E | |
| --- | --- | --- | --- | --- | --- | --- | --- | --- | --- | --- | --- | --- |
|  | r | *p* | r | *p* | r | *p* | r | *p* | r | *p* | r | *p* |
| TP | -0.086 | 0.859 | -0.111 | 0.941 | -0.038 | 0.586 | 0.121 | 0.121 | -0.100 | 0.796 | 0.179 | 0.110 |
| TN | -0.009 | 0.511 | -0.085 | 0.873 | -0.057 | 0.659 | 0.119 | 0.102 | -0.088 | 0.771 | -0.120 | 0.829 |
| acreage | -0.129 | 0.979 | -0.108 | 0.938 | 0.079 | 0.228 | 0.082 | 0.199 | 0.015 | 0.372 | -0.084 | 0.709 |
| PO_4_^3-^ | 0.034 | 0.279 | -0.114 | 0.963 | 0.109 | 0.120 | -0.110 | 0.885 | 0.142 | 0.097 | 0.185 | 0.103 |
| biomass | - | - | - | - | 0.050 | 0.289 | -0.129 | 0.963 | -0.034 | 0.571 | -0.012 | 0.483 |
| isolation | -0.054 | 0.831 | -0.078 | 0.947 | -0.063 | 0.811 | -0.066 | 0.856 | -0.076 | 0.732 | -0.157 | 0.942 |
